# Genetic dissection of transcript, metabolite, growth and wood property traits in an F2 pseudo-backcross pedigree of *Eucalyptus grandis x E. urophylla*

**DOI:** 10.1186/1753-6561-5-S7-O7

**Published:** 2011-09-13

**Authors:** Maria Magdalena van Dyk, Anand R K Kullan, Eshchar Mizrachi, Charles A Hefer, L’Zanne Jansen van Rensburg, Timothy J Tschaplinski, Katherine C Cushman, Nancy E Engle, Gerald A Tuskan, Nicoletta Jones, Arnulf Kanzler, Alexander A Myburg

**Affiliations:** 1 University of Pretoria, Department of Genetics, Forestry and Agricultural Biotechnology Institute (FABI), South Africa; 2 Biosciences Division, Oak Ridge National Laboratory, USA; 3 Sappi Forests Research, South Africa

## Background

*E. grandis *is used extensively for the production of pulp and paper due to its rapid growth, good form and ease of vegetative propagation. *E. urophylla* exhibits tolerance to fungal diseases that limit the growth of *E. grandis* in tropical and subtropical regions. Interspecific hybrids of these two species are, therefore, commonly used to produce fast-growing, disease tolerant hybrids for clonal eucalypt plantations in tropical and subtropical regions (e.g., South Africa, Congo and Brazil). These hybrids often exhibit superior growth and wood quality compared to the pure species, but the underlying genetic basis of the observed hybrid superiority remains unclear. Phenotypic variation observed in interspecific mapping populations has been used to identify QTLs in several genetic linkage studies in *Eucalyptus*. However, QTL intervals have generally been wide (20 to 30 cM) and may include several hundred genes. To bridge the gap between fine mapping and QTL validation studies, the expression levels of genes and metabolites in individuals from the segregating population can be treated as quantitative traits and used for eQTL and mQTL mapping, respectively. Co-localization of wood property, expression and metabolite QTLs will facilitate the identification of positional candidate genes and other components of regulatory networks underlying phenotypic variation.

## Methods

To identify genetic factors controlling growth and wood property traits in *Eucalyptus*, an F_2_ pseudo-backcross mapping family derived from a cross between an F_1_ hybrid (GUSAP1, *E. grandis*x*E. urophylla*, Sappi Forest Research) and an *E. urophylla* parent (USAP1), consisting of 555 individuals, was used for genetic linkage map construction using microsatellite and DArT markers. Phenotypic trait assessment included physical measurements of tree diameter and wood density performed on 319 three-year-old individuals. Klason (acid-soluble & -insoluble) lignin and cell wall sugar content were determined for a selection of 100 backcross progeny and used for near-infrared analysis (NIRA) calibration. NIRA predictions for glucose, xylose, arabinose, cellulose and total lignin content, as well as pulp yield were made for all 315 individuals. Total lignin and S:G ratios were also separately measured for the 315 individuals. Immature xylem tissues, collected from 192 backcross progeny, were used for metabolite profiling (ORNL, Oak Ridge, TN) and Illumina mRNA-Seq (PE50, BGI Americas) quantification of transcript levels.

## Results and discussion

Parental genetic linkage maps were constructed for the F_1_ hybrid (GUSAP1, *E. grandis* x *E. urophylla*) and the *E. urophylla* backcross parent (USAP1, Kullan et al. submitted).An integrated parental linkage map, constructed using the cross pollinator (CP) population type in JoinMap^®^4, was used for QTL mapping as this allows for the detection of the effect of alleles inherited from a single parent and the interaction between the alleles inherited from both parents. In addition to more than 50 growth and wood property trait QTLs (physical measurements, NIRA predictions and wood chemistry traits) and more than 80 metabolite QTLs (mQTLs), representing variation in 22 known metabolites, we will present progress made towards the mapping of gene expression QTLs (eQTLs) based on mRNA-Seq profiling of the first 96 backcross individuals.

## Conclusions

High-density genetic linkage maps, as constructed during this investigation, enable the accurate identification of genomic regions harbouring QTLs, mQTLs and eQTLs. This is an on-going investigation in which we will integrate different genomics data with the aim of dissecting regulatory networks underlying growth and development in *Eucalyptus*.

